# Bone augmentation using a new injectable bone graft substitute by combining calcium phosphate and bisphosphonate as composite—an animal model

**DOI:** 10.1186/s13018-015-0263-z

**Published:** 2015-07-25

**Authors:** Carsten W. Schlickewei, Georg Laaff, Anne Andresen, Till O. Klatte, Johannes M. Rueger, Johannes Ruesing, Matthias Epple, Wolfgang Lehmann

**Affiliations:** Department of Trauma, Hand and Reconstructive Surgery, University Medical Center Hamburg-Eppendorf, Martinistrasse 52, 20246 Hamburg, Germany; Inorganic Chemistry and Center for Nanointegration Duisburg-Essen (CeNIDE), University of Duisburg-Essen, Universitaetsstr. 5-7, 45117 Essen, Germany

**Keywords:** Bone graft substitute, Critical size defect, Bisphosphonate, Calcium phosphate, Alendronate

## Abstract

**Objective:**

The aim of this study was to create a new injectable bone graft substitute by combining the features of calcium phosphate and bisphosphonate as a composite bone graft to support bone healing and to evaluate the effect of alendronate to the bone healing process in an animal model.

**Material and method:**

In this study, 24 New Zealand white rabbits were randomly divided into two groups: a calcium phosphate alendronate group and a calcium phosphate control group. A defect was created at the proximal medial tibia and filled with the new created injectable bone graft substitute calcium phosphate alendronate or with calcium phosphate. Healing process was documented by fluoroscopy. To evaluate the potential of the bone graft substitute, the proximal tibia was harvested 2, 4, and 12 weeks after operation. Histomorphological analysis was focused on the evaluation of the dynamic bone parameters using the Osteomeasure system.

**Results:**

Radiologically, the bone graft materials were equally absorbed. No fracture was documented. The bones healed normally. After 2 weeks, the histological analysis showed an increased new bone formation for both materials. The osteoid volume per bone volume (OV/BV) was significantly higher for the calcium phosphate group. After 4 weeks, the results were almost equal. The trabecular thickness (Tb.Th) increased in comparison to week 2 in both groups with a slight advantage for the calcium phosphate group. The total mass of the bone graft (KEM.Ar) and the bone graft substitute surface density (KEM.Pm) were consistently decreasing. After 12 weeks, the new bone volume per tissue volume (BV/TV) was still constantly growing. Both bone grafts show a good integration. New bone was formed on the surface of both bone grafts. The calcium phosphate as well as the calcium phosphate alendronate paste had been enclosed by the bone. The trabecular thickness was higher in both groups compared to the first time point.

**Conclusion:**

Calcium phosphate proved its good potential as a bone graft substitute. Initially, the diagrams seem to show a tendency that alendronate improves the known properties of calcium phosphate as a bone graft substitute. The composite graft induced a good and constant new bone formation. Not only the graft was incorporated into the bone but also a new bone was formed on its surface. But we could not prove a significant difference between the grafts. Both implants proved their function as a bone graft substitute, but the bisphosphonate alendronate does not support the bone healing process sufficiently that the known properties of calcium phosphate as a bone graft substitute were improved in the sense of a composite graft. In this study, alendronate used as a bone graft in a healthy bony environment did not influence the bone healing process in a positive or negative way.

## Background

The treatment of critical size bone defects still represents one of the most challenging problems for orthopedic and trauma surgeons. Despite intense attempts to develop new bone graft substitutes, the autologous bone graft still constitutes the golden standard for the treatment of critical size bone defects [1, 2]. Nevertheless, the application and the harvesting of autologous bone are limited due to the restricted availability and the possible donor site morbidity like nerve lesions, chronic pain, disease transmission, superficial infections, or hematoma [3–7]. These restrictions underscore the need to develop new effective bone graft substitutes.

Calcium phosphates represent one of the best-known and clinically established bone grafts [8–11]. Since more than a century, different investigations have proven their potential as a bone graft substitute [12]. Calcium phosphate stimulates bone healing by its osteoconductivity, biocompatibility, and biodegradation [8, 9, 11].

Today, the structure of bone is regularly influenced by medication [13]. The number of elderly patients, which use bisphosphonates as protection against osteoporosis, is steadily rising. Additionally, bisphosphonates are commonly used as effective therapeutic drugs for other bone diseases like the Paget disease or metastatic bone lesions. There, bisphosphonates are used to inhibit the mineralization of the bone substance as well as the bone resorption by suppressing the osteoclast activity. Despite the increasing clinical use of bisphosphonates, there are only limited data available that analyze the impact of bisphosphonate on the bone healing process during an acute fracture situation or as a bone graft in a bony defect. While some studies primarily reported a delayed callus remodeling, no adverse effect on fracture healing was reported [14–17]. Some of these studies even reported a secondary improvement of the bone mineral content after delayed callus remodeling over time [16, 18, 19]. Other studies described positive effects of bisphosphonates on the bone healing process. They reported an enhanced strength and an improved tissue volume [17, 20–22]. Kakar even reported the successful treatment of a delayed union (7 months) after open tibia fracture with the application of two doses of bisphosphonate to a 9-year-old child [23]. However, osteonecrotic actions of bisphosphonates have also been reported [24].

These results indicate that bisphosphonates may be able to enhance fracture healing and also to improve common bone substitutes [25], in line with reports on calcium phosphate/bisphosphonate composites for orthopedic application [26–30].

While the most studies investigated the impact of bisphosphonates in an osteoporosis model and focused thereby on the effect on bone resorption, we wanted to examine the use of calcium phosphate and bisphosphonate as a composite bone graft implanted into a large bony defect in a healthy bony environment. The aim was to evaluate the drug effect of alendronate as a bone graft substitute and not the already proven positive effect of alendronate on bone resorption in an osteoporotic model.

The idea of this study was to create a new injectable bone graft substitute by combining the properties of calcium phosphate and bisphosphonate as composite bone graft implanted in a healthy bony environment. The use of bisphosphonates as bone graft substitute and the direct impact of bisphosphonates on the bone healing process in a large bone defect were investigated.

## Materials and methods

### Calcium phosphate

Calcium phosphates represent a well-known group of bone graft substitutes [8–11]. One of the most characteristic variables of calcium phosphate (Ca/P) is the molar ratio of calcium to phosphate. This ratio reaches from 0.5 in monocalcium phosphate (Ca(H_2_PO_4_)_2_) to 2.0 in tetracalcium phosphate (Ca_4_(PO_4_)_2_O). Thereby, the molar ratio of calcium to phosphate determines the water solubility [31]. A lower ratio of calcium to phosphate usually means higher water solubility under physiological pH conditions. Monocalcium phosphate shows such good water solubility that it does not occur in biological hard tissue like bone. Hydroxyapatite (Ca_5_(PO_4_)_3_(OH)) has specific biological relevance with a calcium to phosphate ratio of 1.67. The water solubility amounts 3.10^−4^ g L^−1^ at 25 °C [32]. Hydroxyapatite can be produced in precipitation reactions of a calcium salt solution and a phosphate solution in a neutral or alkaline pH range. Hydroxyapatite represents the main inorganic component of the bone with enclosed ions like fluoride, magnesium, carbonate, or hydrogen phosphate [33]. The bone itself represents a living tissue that is continuously undergoing remodeling and repair due to the activity of osteoclasts and osteoblasts [34].

For this study, nanoparticulate calcium phosphate as a bone graft substitute was chosen. Nanoparticulate calcium phosphate was prepared by a precipitation reaction [35], and a secondary stabilization with carboxymethylcellulose (CMC) was performed as described earlier.

### Bisphosphonate

**Fig. 1:**
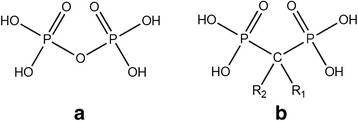
Structural formulae of **a** pyrophosphate P-O-P and **b** bisphosphonate P-C-P

Bisphosphonates represent a chemically stable synthetic analog to pyrophosphate. By the exchange of the oxygen in the hydrolysable phosphorus-oxygen-phosphorus (P-O-P) structure of pyrophosphate, bisphosphonates show resistance to enzymatic and chemical hydrolysis as the phosphorus-carbon bond is not subject to hydrolysis [36]. Concerning the chemical formula, the residues R1 and R2 allow to prepare different bisphosphonates (Fig. 1).

Despite diverse fields of usage, medicine constitutes the most important application area for bisphosphonates. They are used in the treatment of most diverse metabolic bone or calcium diseases with a high bone resorption (e.g., osteoporosis) and in the treatment of osseous metastasis or fibrous dysplasia. Bisphosphonates inhibit bone resorption after intake and deposition to the mineral bone surface. They inhibit the mineralization of the bone substance as well as bone resorption by suppression of osteoclast activity.

**Fig. 2:**
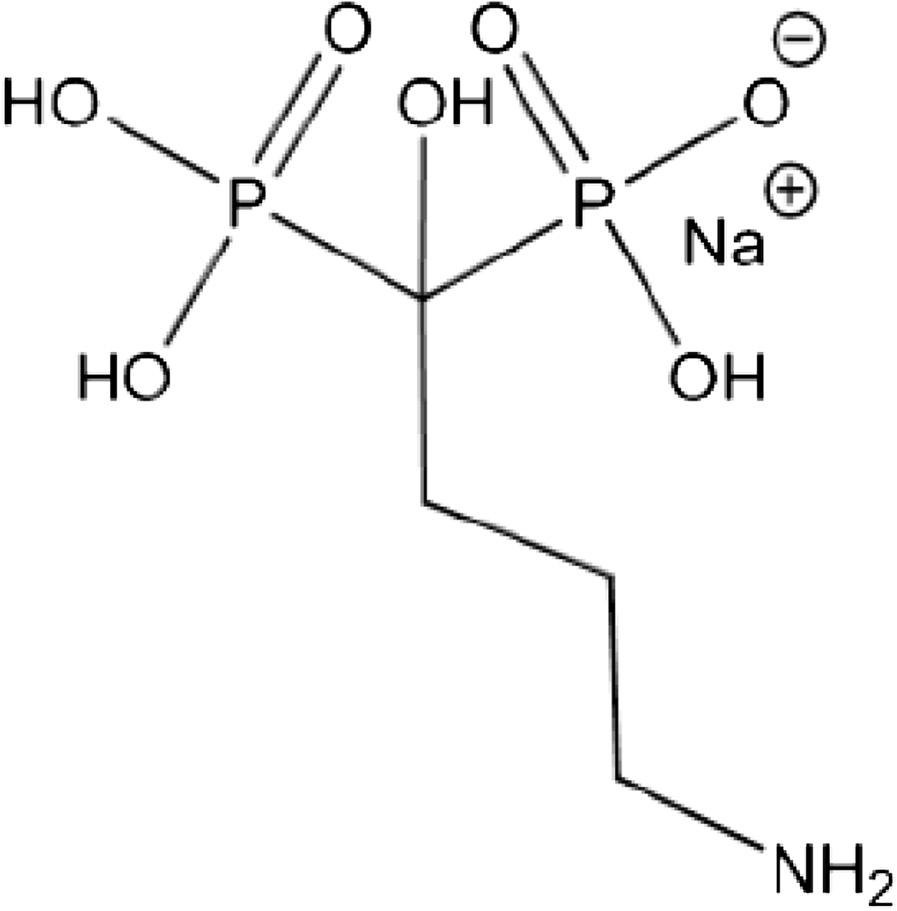
Structural formula of bisphosphonate

In this study, the often applied bisphosphonate sodium alendronate was chosen to augment the calcium phosphate. The residues R1 and R2 consist of -OH and -(CH_2_)_3_NH_2_ (Fig. 2).

Due to the fact that the literature does not give a precise answer regarding the influence of bisphosphonates as a bone graft substitute on the bone healing process in a healthy bony environment, this study is of legitimate interest.

Our objective was to create an injectable paste consisting of nanoparticulate calcium phosphate and sodium alendronate.

### Bone graft substitute

**Fig. 3:**
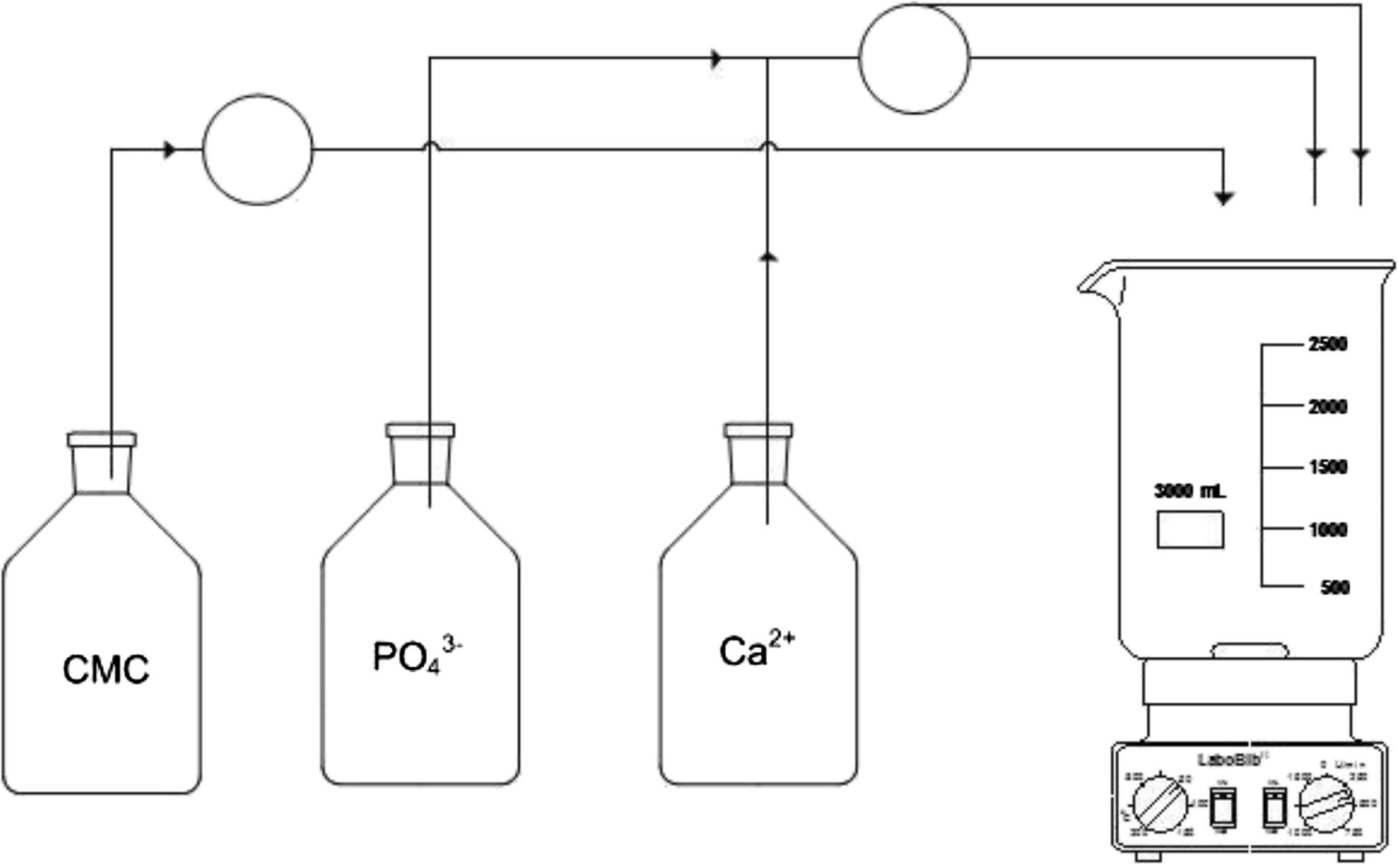
The preparation of the nanopaste, i.e., a gradual mixing of aqueous solutions of *CMC*, di-ammonium hydrogen phosphate, and calcium-*L*-lactate

CMC-stabilized nanoparticulate calcium phosphate was prepared by continuously mixing aqueous solutions of calcium-*L*-lactate pentahydrate solution (Merck, p.a., 5.54 g L^−1^), *di*-ammonium hydrogen phosphate (Fluka, >99 %; 1.426 g L^−1^) and CMC (Sigma-Aldrich, 90 kDa; 2 g L^−1^) (see ref. [37] for details) (Fig. 3).

**Fig. 4:**
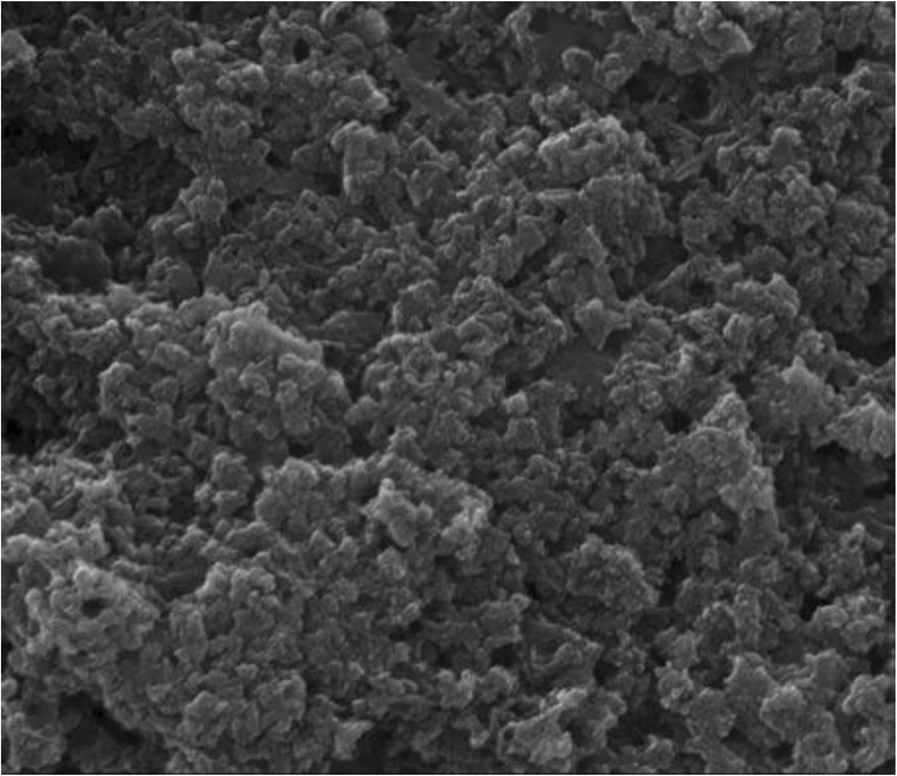
Scanning electron micrograph of autoclaved and freeze-dried nano-calcium phosphate

After five times centrifugation with 5000 rpm (4700 g) and redispersion in water to remove soluble by-products, the dispersion was autoclaved for 20 min at 121 °C. The dispersion was frozen in liquid nitrogen and lyophilized at −7 °C and 0.31 mbar for 4 days. The lyophilized calcium phosphate was stored at −20 °C (Fig. 4).

**Fig. 5:**
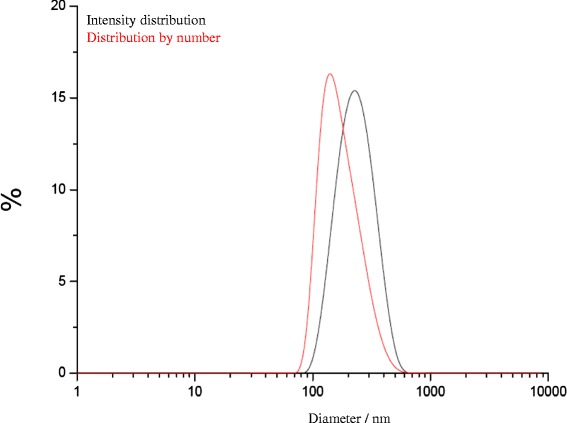
Intensity distribution and distribution by number of CMC-stabilized calcium phosphate nanoparticles

**Fig. 6:**
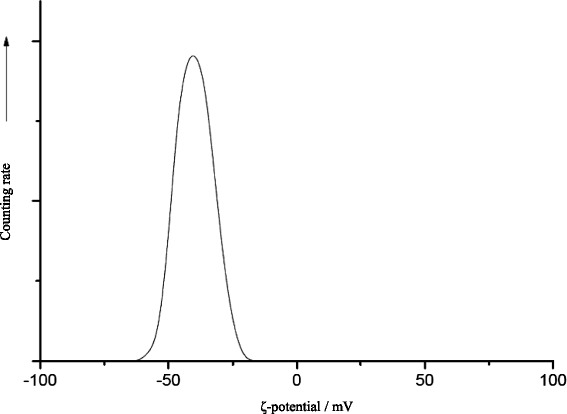
Zeta-potential (*ζ-potential*) of the CMC-stabilized calcium phosphate nanoparticles

**Fig. 7:**
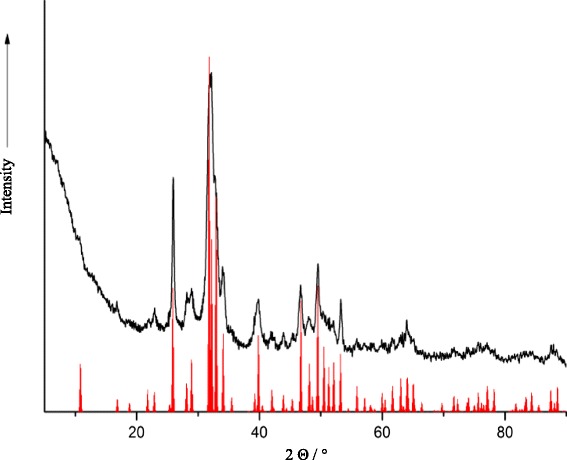
X-ray powder diffractometry of the CMC-stabilized calcium phosphate nanoparticles (*black*) and of hydroxyapatite according to the literature (JCPDS #84-1998) (*red*)

**Fig. 8:**
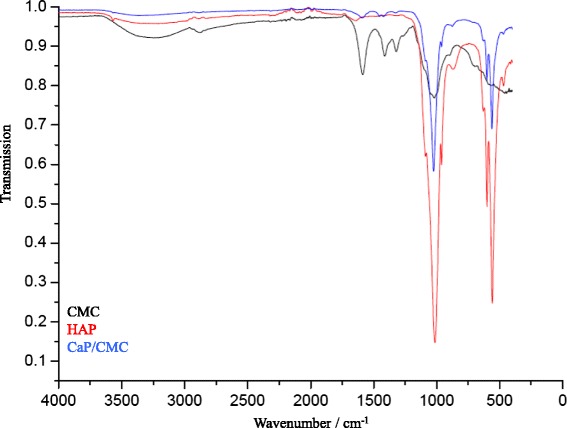
Infrared spectra of carboxymethylcellulose (CMC), hydroxyapatite (HAP), and CMC-stabilized calcium phosphate nanoparticles (CaP/CMC)

The CMC-stabilized calcium phosphate was characterized by dynamic light scattering (Fig. 5), ζ-potential measurement (Fig. 6), X-ray powder diffraction (Fig. 7), and infrared spectroscopy (Fig. 8). The average diameter of the particles was 110 ± 30 nm. Structurally, the particles consist of nanocrystalline hydroxyapatite.

For the operation and filling of the bone defect in the tibia of New Zealand white rabbits, paste of the lyophilized CMC-stabilized calcium phosphate mixed with either 55 % sterile water (control group) or 55 % aqueous alendronate sodium trihydrate solution (2 g L^−1^; alendronate group) was prepared immediately before the injection into the defect.

### Study design

Twenty-four New Zealand white rabbits were included into this pre-clinical study. To ensure skeletal maturity, only rabbits 6 month old or older were chosen. The study design protocol was approved by the Ethics Committee of the University of Hamburg and by the Department for Health and Consumer Protection of the City of Hamburg (reference number of ethical approval 113/11).

The rabbits were housed and supplied under standardized conditions by the Animal Facility Care Unit of the University Hospital Hamburg-Eppendorf. The animals had unlimited access to water and soft chow. At all times, the national guidelines for care and use of laboratory animals in Germany were observed.

After delivery, the New Zealand white rabbits were adapted to the new facility for at least 2 weeks. Preoperatively, the animals were randomly divided into two groups. Twelve rabbits each were randomly chosen for the calcium phosphate control group (no alendronate) and the calcium phosphate alendronate group, respectively.

Before surgery, each animal was narcotized. Anesthesia was performed using 0.1 mg/kg Atropin (Atropinum Sulfuricum®, Eifelfango, Bad Neuenahr-Ahrweiler), 6 mg/kg Xylazin (Rompun®, Bayer, Leverkusen), and 60 mg/kg Ketamin (Ursotamin®, Serumwerk Bernburg).

Afterwards, the rabbits were transferred back into their cage until the animals were certainly narcotized. Additionally, an anesthetic mask was placed over the head of the rabbits and floated with narcotic gas to support the anesthesia.

**Fig. 9:**
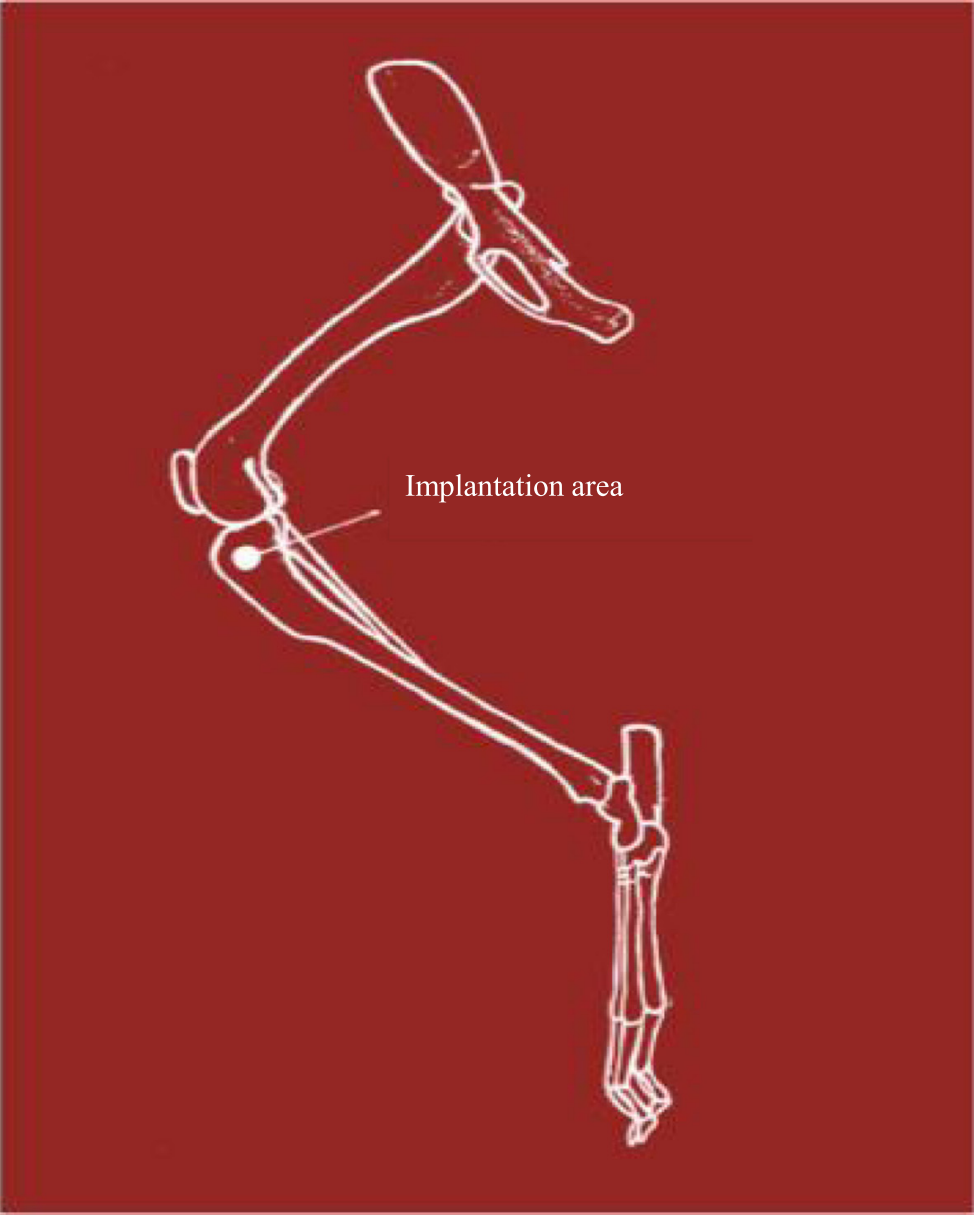
*Implantation area*—the right proximal medial rabbit tibia—approximately 5 mm below the joint line

The selected implantation area was the right proximal medial rabbit tibia, approximately 5 mm below the joint line (Fig. 9). Under anesthesia, the skin was shaved using a hair trimmer. Disinfection was performed repetitively with Cutasept® G (Bode, Hamburg, Germany). After local anesthesia with Scandicain 2 % (AstraZeneca GmbH, Wedel, Germany), a 2-cm skin incision was performed over the medial surface of the proximal right tibia. After splitting the periosteum, a Kirschner wire was placed (approximately 5 mm below the joint line) under fluoroscopy control to determine the right entry point for the DBCS® (Diamond Bone Cutting System, Biomet, Darmstadt, Germany). Under constant rinsing with an aqueous NaCl solution (0.9 %), a monocortical unilateral bony defect with an 8.1-mm diameter and 6 mm length was placed at the proximal medial tibia surface using the DBCS® system. The defects were regularly flushed with the saline solution (NaCl 0.9 %). Afterwards, the bone defects were filled with the appropriate amount of the injectable bone graft material.

**Fig. 10:**
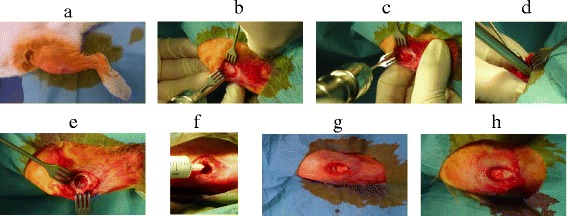
**a** Disinfection of the rabbit tibia. **b** Dissection up to the bone. **c** Insertion of the Kirschner wire. **d** Drilling of the bone defect using the DBCS®. **e** Inspection of the medial proximal bone defect in the rabbit tibia. **f** Injection of the bone graft. **g** Bone defect filled with calcium phosphate paste (control). **h** Bone defect filled with calcium phosphate/alendronate paste

The preparation of the bone graft paste was carried out during surgery as described above. The bone graft material with a consistency similar to toothpaste was injected into the defect with a syringe. Afterwards, the wound was sealed with a nonabsorbable monofilament Ethilon® 3/0 suture (Ethicon INC., Johnson & Johnson, NJ, USA) (Fig. 10).

To minimize the bleeding, a pressure bandage was placed over the wound for 10 min. After surgery, the animals were monitored until the end of anesthesia and then returned to their original staples. All animals were immediately treated with analgesia. All rabbits were able to move freely under full weight bearing as tolerated.

To evaluate the potential of the injectable nanoparticulate calcium phosphate paste with and without alendronate for a new bone formation, the New Zealand white rabbits were sacrificed and the proximal tibia was harvested at 2, 4, and 12 weeks after operation (*n* = 4 for each group at each time point).

### Radiographs

The bone healing process was documented by fluoroscopy. X-ray images were taken in two planes after the operation and at the different time points 2, 4, and 12 weeks.

### Histological preparation

In order to prepare the rabbit tibiae for histological analysis, the remaining soft tissue was carefully removed from the bone with a scalpel. The tibial plateau was then cut approximately 2 cm proximal the defect, and the shaft 2 cm distal the defect in the transversal/axial plane using a diamond-coated saw (EXAKT, Norderstedt, Germany). Each tibial compound was subsequently cut in the sagittal plane at the level of the anterior tibial crest. After dehydration and infiltration, the preparation specimen were embedded into methylacrylate resin, cut into 5–7-μm sections, and stained with toluidine blue.

### Histological analysis and histomorphometry

The histomorphological analysis was focused on the evaluation of the dynamic bone parameters, i.e., the interaction between soft tissue and the bone graft substitute (KEM). Using the semi-automatic image-analyzing Osteomeasure system (Osteometrics, Atlanta, GA, USA), the following parameters were quantified separately for each specimen in order to draw a comparison between the experimental groups: the intramedullary bone volume per tissue volume (BV/TV, %), the trabecular number (Tb.N, mm), the trabecular thickness (Tb.Th, μm), the trabecular separation (Tb.Sp, μm), the number of osteoblasts per tissue area (N.Ob/T.Ar, mm^−2^), the osteoid volume per bone volume (OV/BV, %), the osteoid surface per bone surface (OS/BS, %), the bone graft substitute per area (KEM.Ar, mm^−2^), and the bone graft substitute surface density (KEM.Pm, mm^−2^).

### Statistical analysis

The statistical results are given as mean ± standard deviation. The variance between the calcium phosphate and the calcium phosphate alendronate group was examined using the unpaired, two-tailed Student’s *t* test. ANOVA was used for comparison of different regions of interest. The assumption of a type I error was set at 5 %.

## Results

### Radiograph evaluation

The postoperative X-ray images documented that the defect areas were completely filled with the corresponding bone graft in both groups. Almost no outflow was observed at 2 and 4 weeks. At the last time point, (12 weeks) only sparsely particles of the different bone grafts could be detected by fluoroscopy. In both groups, the volume of bone graft had decreased over the time points, indicating an ongoing resorption.

The follow-up X-ray images at the different time points documented no fracture. After surgery, the rabbits resumed their normal activity, and no side effects could be documented by fluoroscopy. The detailed analysis of the radiographs showed an external callus formation in all tibiae. Postoperatively, no difference was found. After 2 and 4 weeks, the callus thickness in the lateral X-ray images showed an equal distribution for the control and the alendronate group.

**Fig. 11:**
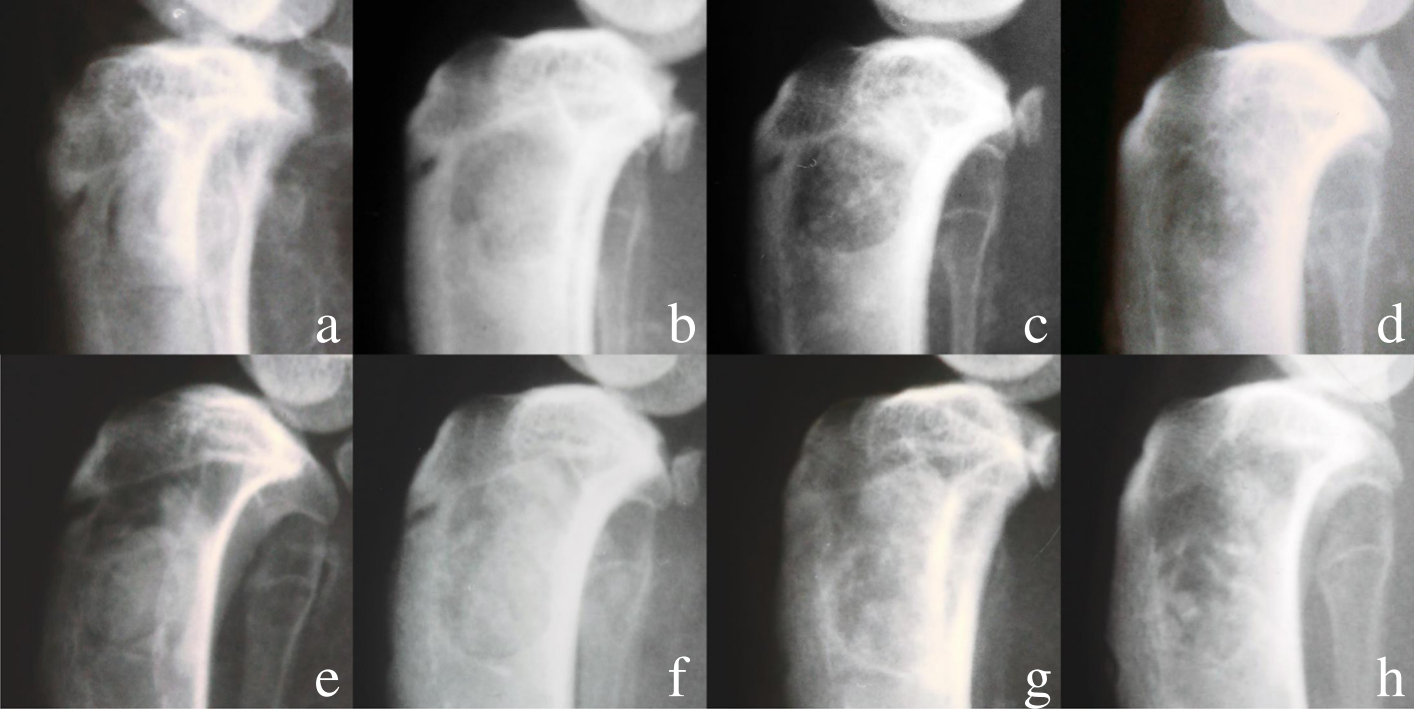
X-rays of the rabbit tibia after the operation and after 2, 4, and 12 weeks. **a**–**d** Calcium phosphate and **e**–**h** Calcium phosphate alendronate

At the final time point (12 weeks), callus thickness showed no visual differences. The bone graft materials were equally absorbed. The bony defects healed normal in all rabbits. Neither any difference between the calcium phosphate paste and the calcium phosphate alendronate paste was demonstrated (Fig. 11, X-Ray 2, 4, and 12 weeks CaP and CaP/alendronate).

### Evaluation of the tissue reaction

#### Results after 2 weeks

**Fig. 12:**
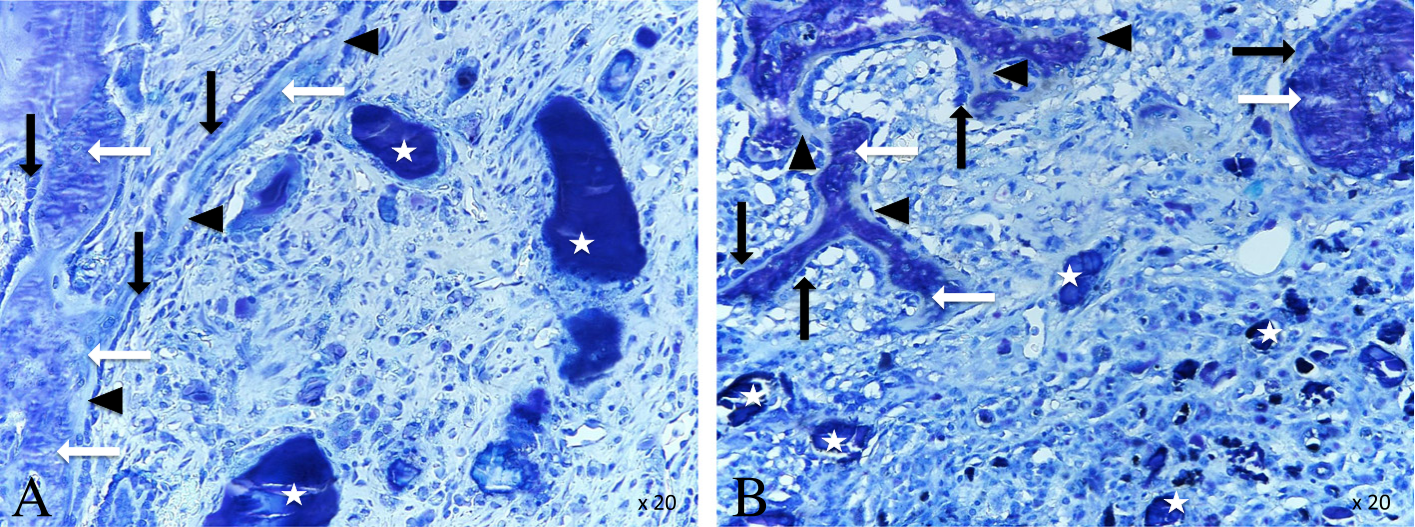
**a** Calcium phosphate 2 weeks: bone graft substitute (*white star*), bone (*white arrow*), osteoid (*black triangle*), and osteoblasts (black arrow). ×20 magnification. **b** Calcium phosphate alendronate 2 weeks: bone graft substitute (*white star*), bone (*white arrow*), osteoid (*black triangle*), and osteoblasts (*black arrow*). ×20 magnification

**Fig. 13:**
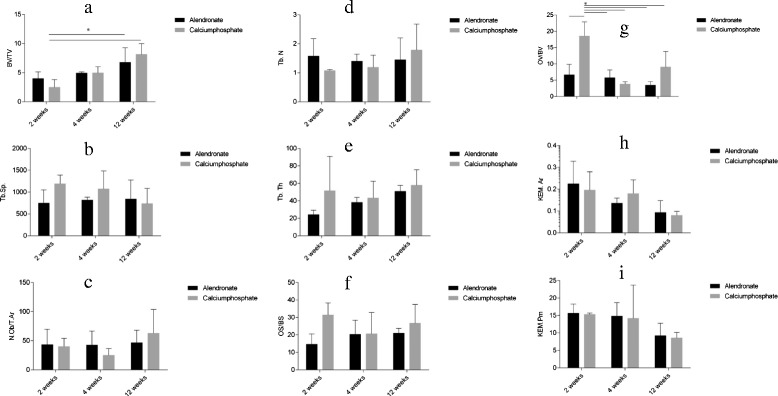
Graphical evaluation of the tissue reaction after 2, 4, and 12 weeks: **a** *BV/TV* bone volume per tissue volume, **b** *Tb.Sp* trabecular separation, **c** *N.Ob/T.Ar* number of osteoblasts per tissue area, **d** *Tb.N* trabecular number, **e** *Tb.Th* trabecular thickness, **f** *OS/BS* osteoid surface per bone surface, **g** *OV/BV* osteoid volume per bone volume, **h** *KEM.Ar* bone graft substitute per area, and **i** *KEM.Pm* bone graft substitute surface density

Two weeks after surgery, the histological analysis showed an increased new bone formation for both analyzed groups. Osteoblasts had started to form a bone tissue by secreting osteoid (Fig. 12). Graphically, the BV/TV slightly suggests a higher value for the calcium phosphate alendronate group. The result can be optically confirmed in the diagrams for the Tb.N and the Tb.Sp (Fig. 13, diagrams). However, the differences between the calcium phosphate and the calcium phosphate alendronate group are not statistically significant. At the first time point, the Tb.Th was equal in both groups, and the N.Ob/T.Ar showed an even distribution.

The OV/BV was significantly higher for the calcium phosphate group. This is also displayed by the OS/BS.

As anticipated, the KEM.Ar and the KEM.Pm showed an equal volume in the area of interest (AOI). In the pasty bone substitutes, mononuclear cells, phagocytes and plasma cells were found. Soft tissue fibers grew in small flaws and uneven areas at the exterior surface of the grafts. The spotted cells were healthy, and no tissue necrosis could be detected.

#### Results after 4 weeks

**Fig. 14:**
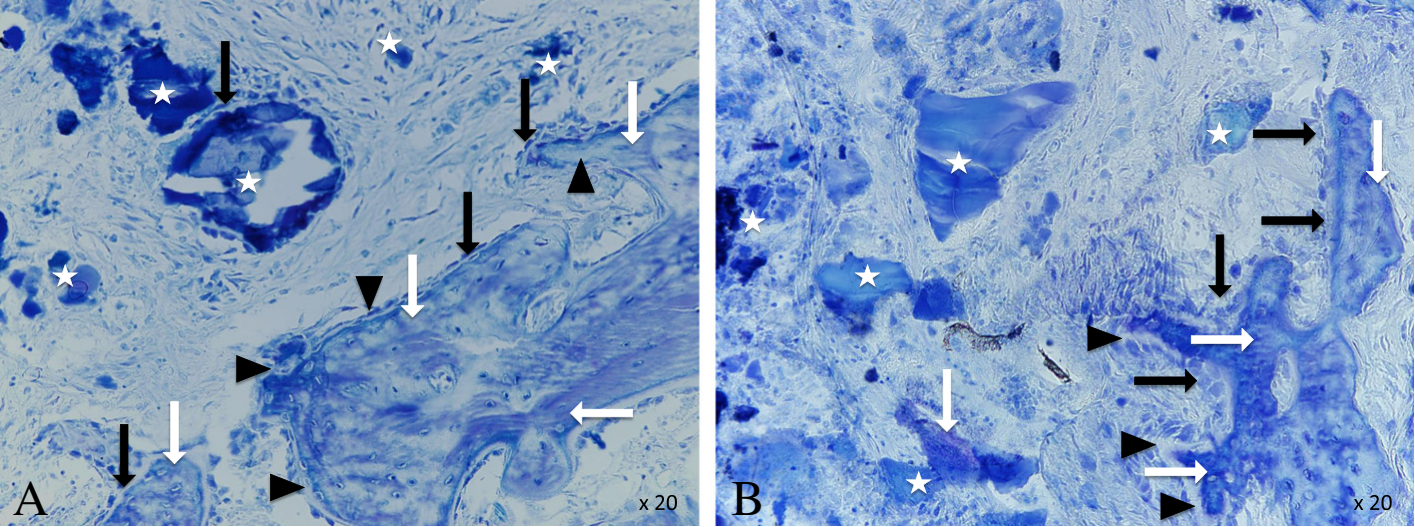
**a** Calcium phosphate 4 weeks: bone graft substitute (*white star*), bone (*white arrow*), osteoid (*black triangle*), and osteoblasts (*black arrow*). ×20 magnification. **b** Calcium phosphate alendronate 4 weeks: bone graft substitute (*white star*), bone (*white arrow*), osteoid (*black triangle*), and osteoblasts (*black arrow*). ×20 magnification

At the second time point, the results for the two different groups were more and more adjusting. Osteoblasts started to attach to the pasty bone graft. More osteoid was produced by the osteoblasts (Fig. 14). The data of the BV/TV showed no difference for new bone formation (Fig. 13, diagrams). The Tb.N and the Tb.Sp adjusted. The Tb.Th increased in comparison to week 2 in both groups with a slight advantage for the calcium phosphate group. The N.Ob/T.Ar in the AOI was slightly declining in both groups but without statistical significance. The increasing mineralization (OV/BV) combined with the growing thickness of the trabeculae implies a maturation of the primarily grown bone.

The KEM.Ar and the KEM.Pm was consistently decreasing. This indicated a good resorption of both bone grafts. No necrosis could be detected. In the AOI, no foreign body reactions were found.

Compared with the first time point, the ingrowth of soft tissue at the outer surface of the grafts had increased equally for both investigated groups.

#### Results after 12 weeks

**Fig. 15:**
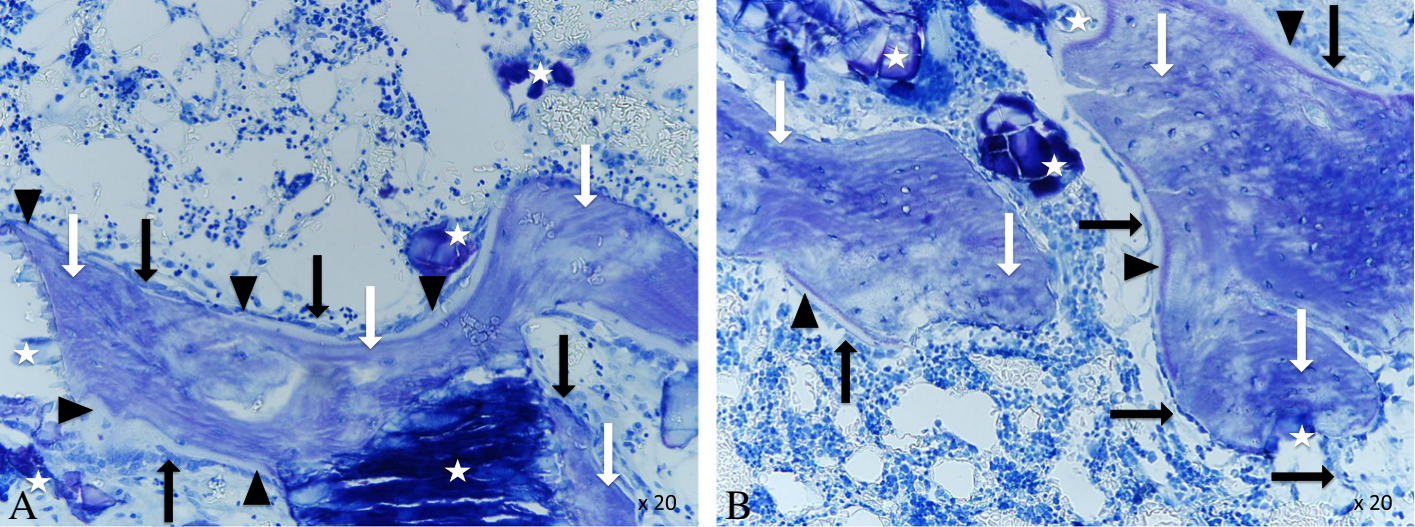
**a** Calcium phosphate 12 weeks: bone graft substitute (*white star*), bone (*white arrow*), osteoid (*black triangle*), and osteoblasts (*black arrow*). ×20 magnification. **b** Calcium phosphate alendronate 12 weeks: bone graft substitute (*white star*), bone (*white arrow*), osteoid (*black triangle*), and osteoblasts (*black arrow*). ×20 magnification

12 weeks after the surgery, the BV/TV was constantly growing (Fig. 13 (diagrams)). Both bone grafts show a good integration. New bone was formed on the surface of both bone grafts. The calcium phosphate as well as the calcium phosphate alendronate paste had been enclosed by bone (Fig. 15). This indicates good osteoconductive properties for the two bone grafts. Calcium phosphate seemed to induce slightly better bone growth after 12 weeks. The tendency was confirmed by the Tb.N and the N.Ob/T.Ar. However, in all diagrams, no statistical significance was found. The trabecular thickness was higher in both groups compared to the first time point. This indicated an ongoing bone maturation, but no significant difference was found when comparing the calcium phosphate group with the calcium phosphate alendronate group. The Tb.Sp was equal. Compared to the first time point, the total mass of the two different KEM.Ar and the KEM.Pm were significantly reduced. Nevertheless, the volume of reduction was equal for both grafts. No signs for tissue necrosis were found. The surgically applied bone defect was not entirely closed in both groups.

## Discussion

Bisphosphonate therapy reduces the risk of secondary fractures in osteoporotic patients [38, 39]. But the ideal time point to introduce the therapy after a recent fracture has not yet been discovered.

The aim of this study was to evaluate the potential of bisphosphonate alendronate to support bone healing in addition to calcium phosphate as a composite bone graft and to evaluate the effect of alendronate to the bone healing process in a healthy bony environment.

Both pasty bone grafts were easy to insert into the bony defect. The injectable paste allowed a secure application during the surgery. During the application, no thermal injury due to missing heat generation occurred. The calcium phosphate as well as the calcium phosphate alendronate bone graft showed a stable texture. The pasty bone grafts did not leak into the surrounding tissue and were incorporated very well into the bone. Both bone grafts proved their osteoconductive properties together with a good biocompatibility. A non-irritated bone healing process was documented for each animal at each time point. The bone grafts were integrated well into the surrounding bone. A new bone was formed on the surface of the bone grafts, underscoring their osteoconductive properties. Over the time, both grafts degraded constantly as assumed.

In accordance with other studies, calcium phosphate proved its good potential as a bone graft substitute [8–11]. Soft tissue fibers attached at the outer surface of the bone graft but did not enter or grow into the bone defect.

Initially, the diagrams seem to show a tendency that alendronate improves the known properties of calcium phosphate as a bone graft substitute. The composite graft induced a good and constant new bone formation. The graft was not only incorporated into the bone, but also a new bone was formed on the surface of the graft. But we could not prove a significant difference between the calcium phosphate paste and the calcium phosphate alendronate paste.

The direct implantation of alendronate as a composite into the bony defect could influence the bioavailability, delivery, and drug reaction as Peter et al. already assumed [40].

While different studies investigated the impact of bisphosphonates in an osteoporotic model and proved positive results, we implanted the composite into a healthy bony environment [40–42]. Some studies explain the increase of bone mass after the application of bisphosphonates due to the decrease of bone turnover and not as a consequence of an enhanced bone healing [43–45]. In addition to these results and to the assumption of Peter et al. [40], Verron et al. described the development of an injectable composite bone graft of calcium phosphate and bisphosphonate in an osteoporotic sheep model [46]. He demonstrated that bisphosphonate-loaded cement positively influenced the microarchitecture of the adjacent bone. The effect was relative to the distance from the bone graft and indicated an in situ effect of the bisphosphonate [46]. This refuted the assumptions of Peter et al. who indicated a negative influence on the bioavailability and drug reaction of alendronate after direct implantation into the bony defect. In this study, the missing influence of alendronate to the microarchitecture of the adjacent bone is probably a consequence of the healthy bony environment surrounding the defect. We assume that the positive effect of bisphosphonates in osteoporotic models is produced by a decrease of bone turnover and not as a consequence of enhanced bone healing [43–46]. To verify this, a study should be designed that compares the implantation of a calcium phosphate alendronate composite in an osteoporotic and a healthy bone model.

In our study, both implants proved their function as bone graft substitute, but the presence of alendronate had neither a positive nor a negative effect on the bone healing process at the different time points. Due to the study design, we were not able to assess the impact of a long-term alendronate therapy to the bone healing process.

Bone healing and callus formation were assessed by fluoroscopy. The X-ray images documented that all bone defects in the tibia heads healed with an external osseous callus formation. No fracture occurred during the observation time. Neither could we prove a delayed callus formation [47] nor did we observe a much larger callus formation as Li found for bisphosphonates in his long-bone fracture model in rats [14]. In Li’s study, the callus formation in the calcium phosphate alendronate group was not inhibited. We cannot report any increase of complications. In this study, callus thickness showed no visual differences. When comparing the fluoroscopy pictures at the different time points, alendronate did not influence the bone healing process.

As a limitation for this study, we have to cite that we only used one dose of alendronate for the preparation of the composite. Peter et al. already proved the dose effect of bisphosphonates on the bone volume fraction in an osteoporotic rat model [40]. We recommend additional studies that compare different alendronate concentrations to evaluate the dose effect.

## Conclusions

In summary, alendronate had no positive or negative benefit as a bone graft substitute regarding this study. After injection together with calcium phosphate into the bone defect in a healthy bony environment, it did not lead to an increased callus formation. In this study, alendronate did not influence the bone healing process at the different time points.

Due to our outcomes, the bisphosphonate alendronate composite does not support the bone healing process in a healthy bony environment sufficiently that the known properties of calcium phosphate as a bone graft substitute were improved in the sense of a composite graft. We assume that the missing influence of alendronate to the microarchitecture of the adjacent bone is probably a consequence of the healthy bony environment surrounding the defect. To verify this, a study should be designed that compares the implantation of a calcium phosphate alendronate composite in an osteoporotic and a healthy bone model.

Regarding our results and the collected data of different studies [48–50], it appears to be safe to start with an initial alendronate therapy directly after a recent bone fracture. Due to the study design, we were not able to assess the impact of a long-term alendronate therapy to the bone healing process. We suggest additional studies to evaluate the duration of action of a calcium phosphate alendronate composite bone graft.

## Abbreviations

AOI: area of interest
BV/TV: bone volume per tissue volume
Ca(H_2_PO_4_)_2_: monocalcium phosphate
Ca/P: calcium phosphate
Ca_4_(PO_4_)_2_O: tetracalcium phosphate
Ca_5_(PO_4_)_3_(OH): hydroxyapatite
CMC: carboxymethylcellulose
DBCS: diamond bone cutting system
KEM: bone graft substitute
KEM.Ar: bone graft substitute per area
KEM.Pm: bone graft substitute surface density
N.Ob/T.Ar: number of osteoblasts per tissue area
OS/BS: osteoid surface per bone surface
OV/BV: osteoid volume per bone volume
P-O-P: phosphorus-oxygen-phosphorus
Tb.N: trabecular number
Tb.Sp: trabecular separation
Tb.Th: trabecular thickness
